# The allelic distribution of -308 Tumor Necrosis Factor-alpha gene polymorphism in South African women with cervical cancer and control women

**DOI:** 10.1186/1471-2407-6-24

**Published:** 2006-01-26

**Authors:** Vandana A Govan, Debbie Constant, Margaret Hoffman, Anna-Lise Williamson

**Affiliations:** 1 Division of Medical Virology, Institute of Infectious Diseases and Molecular Medicine, University of Cape Town, Observatory, Cape Town, South Africa; 2 School of Public Health, Faculty of Health Sciences, University of Cape Town, Observatory, Cape Town, South Africa; 3 National Health Laboratory Services, Observatory, Cape Town

## Abstract

**Background:**

Cervical cancer is due to infection with specific high-risk types of human papillomavirus (HPV). Although the incidence of genital HPV infection in various population groups is high, most of these regress without intervention. Investigating genetic host factors and cellular immune responses, particularly cytokines, could help to understand the association between genital HPV infection and carcinogenesis. The tumor necrosis factor alpha (TNF-α) cytokine plays an important role in all stages of cervical cancer and has the ability to induce the regression of human tumors. Therefore the aim of the study was to investigate the allelic distribution of -308 TNF-α gene polymorphism in South African women with cervical cancer compared to control women.

**Methods:**

Included in our study were women with histologically proven cancer of the cervix (n = 244) and hospital-based controls (n = 228). All patients and controls were from mixed race and black population groups in South Africa. The detection of a bi-allelic -308 (A/G) polymorphism in the promoter region of TNF-α was investigated using the amplification refractory mutation system-polymerase chain reaction (ARMS-PCR) technique. The distributions of the allelic frequencies were stratified in both patients and controls into two South African ethnic population groups.

**Results:**

In this study we observed no association between the distribution of -308 TNF-α polymorphism and the risk of developing cervical cancer even after combining the data from the two ethnic populations (X^2 ^= 2.26). In addition, using the chi-squared test we found no significant association between the known risk factors for cervical cancer and the allele distribution of -308 TNF-α. However, the frequency of the rare high-producing allele -308A of TNF-α was significantly lower in the South African population when compared to Caucasians and Chinese population groups.

**Conclusion:**

We demonstrated no association between -308 TNF-α polymorphism and the risk of cervical cancer among two South African ethnic population groups. However, as the distribution of the -308A TNF-α was notably different between the control groups of South Africa and other population groups this result suggests that ethnic disparity may influence the levels of TNF-α produced.

## Background

There is strong epidemiological and experimental data that have demonstrated a definite association of high-risk human papillomavirus (HR-HPV) infection and the development of cervical cancer [1]. Interestingly, although most sexually active women in the normal population have asymptomatic cervical HPV infections, most of these cervical infections are transient, with clearance in 70% to 90% of individuals positive for HPV DNA. In addition, only a small percentage develops long-term HPV infection, which is associated with an increased risk of developing cervical cancer [2]. Thus, an effective host immune response may be an important determinant for the persistence and progression of HPV induced cervical cancer. In particular, cell-mediated immunity (CMI) is important in controlling both HPV infections and HPV-associated neoplasms [3]. CMI is regulated by cytokines that are secreted primarily by T helper (Th) cells and macrophages.

Cytokines play a significant role in the defense against HPV induced infections, modulating viral replication and polarizing the immune response to a Th1 (cellular) or Th2 (humoral) pattern [1]. Th1 cells are immuno-stimulatory and are associated with the clearance of HPV infection and regression of cervical intraepithelial neoplasia [4] Th2 cells are immuno-inhibitory and are capable of stimulating tumor growth [5]. Investigating genetic host factors and immune responses could help to understand the association between genital HPV infection and carcinogenesis [6], as cervical cancer is the second most common female cancer worldwide and the most common cancer in South Africa among women, which continues to be a public health burden. Several candidate gene studies have demonstrated that genetic polymorphisms in cytokine genes contribute to the variation in the levels of cytokine produced and this variation may influence the severity of several infectious diseases [7-9]. Among them the pro-inflammatory cytokine, tumor necrosis factor-alpha (TNF-α) has been of particular interest as it was found to be located in the central major histocompatibility complex (MHC) and a possible genetic correlation between TNF alleles and disease susceptibility was hypothesized [10].

TNF-α is a multifunctional cytokine that was originally identified as a macrophage-derived serum protein that mediates necrosis of solid tumors in vitro and in vivo [11]. In addition, TNF has been shown to mediate carcinogenesis through induction of proliferation, invasion, and metastasis of tumor cells [12]. Furthermore, it was shown that the expression of TNF-α is regulated at the transcriptional level [13] and various polymorphisms within the TNF-α promoter region have been associated with the level of TNF-α produced [10,14,15]. Several investigators have studied the polymorphisms within the TNF-α promoter region to estimate the immune responses to a wide range of cancers [9,16,17] including cervical cancer [18,19]. In particular, the biallelic polymorphism in the promoter region at position -308 relative to the transcriptional start site of the gene, representing a transition from the nucleotides guanine (G) to adenine (A) has been commonly studied. This -308G/A transition affects the expression of TNF-α where the less common -308A allele produces higher levels of TNF-α, while the -308G allele is linked to a reduced TNF-α production [10,20]. Indeed, these observations presented the possibility that tumor development may be associated to the genetic predisposition of the host to produce higher levels of TNF-α. Numerous studies have investigated the association between the effect of TNF-α promoter region and cervical cancer, but the results have been contradictory [9,18,21,22]. Notably, these findings are from different population groups and thus indicate that the variation in TNF-α production may be influenced by the genetic make-up of diverse population groups contributing to a disparity in disease outcome.

Therefore, given the importance of TNF-α in potentially contributing to the progression of cervical cancer with variations in the levels of TNF production in different population groups, we investigated the biallelic polymorphism in the -308 promoter region of the TNF-α gene using the ARMS-PCR methodology and examined its relationship to the development of cervical cancer among two South African ethnic groups.

## Methods

### Patients and control populations

Individuals were selected from a case-control study of cancer of the cervix and related risk factors conducted among colored (mixed racial descent) and black women residing in the Western Cape Province of South Africa, from January 1998 to December 2001 [23]. The Research Ethics Committee, Faculty of Health Sciences, University of Cape Town, South Africa, approved the case-control study. Cases were women with invasive cancer of the cervix attending Gynae-oncology clinics at 2 tertiary hospitals. The controls, who were hospital based, were women with primary diagnosis such as trauma or acute infections that were judged to be independent of contraceptive use and had no history or evidence of cervical disease and were identified at the 2 tertiary hospitals. The controls were series matched to cases for decade age, ethnic group and area of residence. Data on known risk factors and potential confounders were collected using a detailed questionnaire administered by trained nurse interviewers. Papanicoloau (Pap) smears were taken from all control women. Blood samples from randomly selected 244 eligible cervical cancer patients and 228 hospital-based controls were analysed for the -308 polymorphisms in the promoter region of TNF-α.

### DNA extraction

Genomic DNA was extracted from 200 μl of whole blood using the QiAamp Spin Blood Kit (QIAGEN, Valencia, CA) in accordance with manufacturer's instructions.

### Cytokine genotyping

The A and G alleles at position -308 in the promoter region of the TNF-α gene were identified using the amplification refractory mutation system polymerase chain reaction (ARMS-PCR) methodology as previously described [24]. A total of 2 ul (25–100 ng) of DNA was used in each ARMS-PCR reaction. The PCR primer sequences were as follows:

Generic primer (antisense): 5'-tctcggtttcttctccatcg-3'

Primer G, allele1 (sense): 5'-ataggttttgaggggcatgg-3'

Primer A, allele2 (sense): 5'-aataggttttgaggggcatga-3'

The amplified ARMS-PCR products (PCR product size = 184 bp) were identified by gel electrophoresis on 2 % agarose gels stained with ethidium bromide.

### Statistical analysis

Statistical analyses were performed using STATA version 8. The allele frequency of the -308 TNF-α polymorphism was compared between the patients and control groups using the *X*^2 ^test. Unconditional logistic regression was used to estimate odds ratios (OR's) for developing cancer of the cervix in relation to the -308 TNF-α gene polymorphism. These OR's were adjusted for the following confounding factors; ethnic group, 5 year age group, years of education, age at first sexual intercourse, number of sexual partners, urban/rural living, number of Pap smears, injectable/oral contraceptive use, smoking, and parity.

## Results

To evaluate the accuracy of the assigned TNF-α genotypes a total of twenty-five samples were randomly chosen and the results were confirmed by repeating the ARMS-PCR. Notably there were no discrepant results detected when the ARMS-PCR assay was repeated on the samples.

The genotype distribution of the polymorphism at position -308 of the TNF-α promoter region in the cases and controls among the two South African ethnic groups are shown in Table 1. There were no significant differences in the distribution of TNF-α allelic polymorphisms and the risk of developing cervical cancer in the mixed race group, (OR, 2.93; 95% CI, 0.51–16.72) or the black group (OR, 0.87; 95% CI, 0.27–2.76). In addition, when the data was adjusted for potential confounding factors (adjusted for 5 year age group, education, age at first sex, number of sexual partners, number of Pap smears, injectable/oral contraceptive use, smoking, and parity), no significant association was observed (data not shown).

As there were no significant differences in the distribution of the -308 TNF-α alleles in the cervical cancer patients among the two South African ethnic populations, the data was combined to compare allele distributions to other population groups (Table 2). Interestingly, the genotype distribution of the -308 TNF-a genotypes for the South African cancer cases were different to those reported in a Korean population group (homozygous GG 71% vs 90%; and heterozygous AG 26% vs 6%). In addition, the distributions of the -308 TNF-α alleles in the two South African control groups were combined to compare the genotype distribution to other population groups (Table 3). The genotype distribution for the homozygous GG and AA -308 genotypes for the combined South African data were similar to those reported in Zimbabwean [21], Chinese [25]; Italian [26] and Korean [14] populations. However, the distribution of the heterozygous AG genotype was notably different in the Chinese and Korean compared to the South African control group (8% versus 20 %). In addition, there was an increased frequency of the -308 TNF-α A allele and decrease in the G allele in the British Caucasoid group [24] when compared to the South African population group.

## Discussion

Although the Pap smear screening has reduced cervical cancer mortality in developed countries, 35000 women die from this disease every year in the United States and Europe [27]. The current treatments for cervical cancer are invasive, costly, limited, uncomfortable, inefficient and recurrence is a common outcome [28]. There are several prophylactic and therapeutic clinical HPV vaccine trials that are in progress [29,30], which may assist in reducing this disease burden worldwide. However, while these strategies are commendable it is important to investigate other host or viral markers that contribute to the different stages of cervical disease and thus may provide an effective tool for the treatment of cervical cancer. It is accepted that persistent HRHPV-induced infection is the hallmark of cervical cancer.

Indeed, other co-factors such as viral load, lifestyle factors, high parity, smoking, long-term use of contraceptives and other sexually transmitted diseases have also been established as risk cofactors for cervical cancer among women with persistent HPV infections [31]. In addition, several studies have indicated that the polymorphisms in the promoter region of -308 TNF-α may be a contributing factor for the development of cervical cancer [18,21,32]. However, the results of these studies have been contradictory. Thus in the present study we sought to identify the possible link between -308 A/G TNF-α allele polymorphism and the progression of cervical cancer among two ethnic population groups in South Africa. We found no significant differences between the -308TNF-α allele polymorphism and cervical cancer risk among the two South African ethnic population groups. In addition we observed no significant differences in the distribution of the high (AA), medium (AG) or low (GG) genotypes of TNF-α among the South African cervical cancer patients and healthy controls (and in the combined race groups) (*X*^2 ^= 2.26) even when the data were adjusted for potential confounders (data not shown).

The findings in our study are similar to those reported by Stanczuk *et al*., [21], who investigated the association of -308TNF-α gene polymorphism with cervical cancer among a Zimbabwean population [21]. They found no significant differences in the distribution of the high, medium or low producing alleles in the cervical cancer patients and healthy women. However, their study was based on a small sample size and the authors suggested that a larger cohort should be investigated to challenge this association, as their lack of association using a small sample size may have affected the statistical power of the study. Although our study included a large sample size we were unable to confirm this possibility and found no correlation between -308 TNF-α polymorphism and the risk of developing cervical cancer among the South African population. Similar reports were documented by others [9,22,33] and discordant with those previously reported in a population group from the United Kingdom [15]. In their study the authors demonstrated that the polymorphism in -308TNF-α gene significantly increased the susceptibility to cervical intraepithelial neoplasia 1 (CIN 1). However, as it is well-documented that most CIN1 regress spontaneously [34] it would have been beneficial if the authors extended the follow up screening to 36 months and thus the positive association demonstrated by the authors should be regarded with caution. In addition, CIN1 includes very mild to mild dysplasia, which is characterized by 20–25% replacement of the epithelium with immature cells and generally requires no treatment [29]. Furthermore, although in the same study the recruited subjects were age matched the authors did not provide the ethnic background for the population studied [15]. Thus, it is difficult to conclusively confirm or refute the possible ethnic disparity that may have contributed to the association differences observed between our study population and theirs.

Several reports in cervical cancer survival studies have indicated that the higher mortality rate in black populations is explained primarily by the more advanced clinical stage at time of diagnosis [35,36]. It was found that the percentage of early diagnosis among blacks and Hispanics were significantly low for cervical and breast cancer compared to non-Hispanics whites. It was therefore suggested that screening programs should be managed differently for specific ethnic groups as there is a significant disparity in the outcome of disease between ethnic populations. [36]. Indeed, the high incidence of cervical cancer in certain population groups may be due to poor screening programs, a lack of stringent follow-up treatments and lower socioeconomic status in different population groups. In the present study, we compared our cervical cancer patient group with those from other populations (Table 2). Although there was a difference in the distribution of the GG and AG of TNF-α between our study and the Korean population, this result should be taken with caution as the sample size for the Korean population was very small. It would therefore be valuable if the samples size was increased to examine these relationships adequately. Nevertheless, 26% vs 6% is significant. Furthermore, we compared the distribution of our control study group to those of other populations to identifying whether the distribution of the TNF-α gene at position -308 is different in other healthy population groups (Table 3). The distribution of the AATNF-α, GGTNF-α and AGTNF-α genotypes at position -308 in the present control study group were similar to those observed in a Zimbabwean and Italian population and differed from those found in a Caucasian group. In addition, the distribution of the AGTNF-α genotype in the South African population group differed from that found in a Chinese and Korean population. These data highlight the possible variability of cytokine gene frequencies in different population groups and hence influence the disease outcome.

## Conclusion

It is important to identify host or viral genetic markers that may facilitate the risk of developing cervical cancer. In addition, these markers would be useful in providing effective treatments and preventative strategies against HPV-induced infections. However, in this study we found no association between -308 TNF-α gene polymorphism and the risk of developing cervical cancer. Furthermore, our data highlight the need for further studies to clarify the association of ethnic variation and cytokine polymorphism as other potential confounding factors may contribute to the risk of developing cancer of the cervix.

## Competing interests

The author(s) declare that they have no competing interests.

## Authors' contributions

VAG designed the study, carried out the study and drafted the manuscript. DC performed the statistical analysis for the study. MH participated in the design and coordination of the case control study. A-LW participated in the coordination of the study. All authors read and approved the final manuscript.

## Pre-publication history

The pre-publication history for this paper can be accessed here:



## Figures and Tables

**Table 1:** Odds Ratios and 95% confidence intervals (CI) for the development of cervical cancer in relation to -308 TNF-α alleles among the cervical cancer cases and controls in the two South African ethnic population groups.

**Race**	**Genotype**	**CaCx n (%)**	**Controls n (%)**	**OR^1 ^(95%CI)**
**1. All^#^**		**(n = 244)**	**(n = 228)**	
	GG	174 (71)	172 (76)	1.29 (0.49 – 3.37)
	AG	62 (26)	46 (20)	1.80 (0.66 – 4.97)
	AA	8 (3)	10 (4)	1
**2. Mixed race**		**(n = 83)**	**(n = 59)**	
	GG	64 (77)	45 (76)	2.93 (0.51–16.72)
	AG	17 (21)	10 (17)	3.78 (0.58–24.75)
	AA	2 (2)	4 (7)	1
**3. Black**		**(n = 161)**	**(n = 169)**	
	GG	110 (68)	127 (72)	0.87 (0.27–2.76)
	AG	45 (28)	36 (24)	1.25 (0.37–4.21)
	AA	6 (4)	6 (4)	1

^#^All (1) = Mixed race (2) and Black (3) population groups combined

OR^1 ^= crude odds ratio adjusted for ethnic group in the combined ethnic group analysis

**Table 2:** Distribution (%) of -308 TNF-α genotypes in women with cervical cancer among different population groups.

**Genotype**	*South African n = 244 n (%)	^+^**Korea (South) n = 51 n (%)**	^†^**USA n = 127 n (%)**	^§^**Zimbabwe n = 103 n (%)**
**GG**	174 (71)	46 (90)	91 (72)	74 (72)
**AG**	62 (26)	3 (6)	27 (21)	28 (27)
**AA**	8 (3)	2 (4)	9 (7)	1 (1)

*Combined Black and mixed race cancer case groups in this study (There were no differences in the distribution of the -308 TNF-α alleles between the two South African ethnic populations, thus the data was combined to compare genotype distributions to other population groups).

Data reported by; ^+^[14]; ^†^[9]; ^§^[21].

**Table 3:** Distribution (%) of -308 TNF-α genotypes in the combined South African control population groups and other populations.

**Genotype**	*South African n = 228 n (%)	^**China (North) n = 300 n (%)**	^#^**Italy (North) n = 216 n (%)**	^+^**Korea (South) n = 92 n (%)**	^†^**Manchester n = 106 n (%)**	^§^**Zimbabwe n = 101 n (%)**
**GG**	172 (78)	274 (91)	172 (80)	85 (92.4)	65 (61)	81 (80)
**AG**	46 (20)	24 (8)	41 (19)	7 (7.6)	24 (23)	18 (18)
**AA**	10 (4)	2 (0.7)	3 (1)	0	17 (16)	2 (2)

*Combined Black and mixed race control groups in this study (There were no differences in the distribution of the -308 TNF-α alleles between the two South African ethnic populations, thus the data was combined to compare genotype distributions to other population groups).

Data reported by ˆ[25]; ^#^[26]; ^+^[14]; ^†^[24]; ^§^[21].
